# NAP (davunetide) preferential interaction with dynamic 3-repeat Tau explains differential protection in selected tauopathies

**DOI:** 10.1371/journal.pone.0213666

**Published:** 2019-03-13

**Authors:** Yanina Ivashko-Pachima, Maya Maor-Nof, Illana Gozes

**Affiliations:** Elton Laboratory for Molecular Neuroendocrinology, Lily and Avraham Gildor Chair for the Investigation of Growth Factors, Department of Human Molecular Genetics and Biochemistry, Sackler Faculty of Medicine, Sagol School of Neuroscience and Adams Super Center for Brain Studies, Tel Aviv University, Tel Aviv, Israel; New York State Institute for Basic Research, UNITED STATES

## Abstract

The microtubule (MT) associated protein Tau is instrumental for the regulation of MT assembly and dynamic instability, orchestrating MT-dependent cellular processes. Aberration in Tau post-translational modifications ratio deviation of spliced Tau isoforms 3 or 4 MT binding repeats (3R/4R) have been implicated in neurodegenerative tauopathies. Activity-dependent neuroprotective protein (ADNP) is vital for brain formation and cognitive function. ADNP deficiency in mice causes pathological Tau hyperphosphorylation and aggregation, correlated with impaired cognitive functions. It has been previously shown that the ADNP-derived peptide NAP protects against ADNP deficiency, exhibiting neuroprotection, MT interaction and memory protection. NAP prevents MT degradation by recruitment of Tau and end-binding proteins to MTs and expression of these proteins is required for NAP activity. Clinically, NAP (davunetide, CP201) exhibited efficacy in prodromal Alzheimer’s disease patients (Tau3R/4R tauopathy) but not in progressive supranuclear palsy (increased Tau4R tauopathy). Here, we examined the potential preferential interaction of NAP with 3R vs. 4R Tau, toward personalized treatment of tauopathies. Affinity-chromatography showed that NAP preferentially interacted with Tau3R protein from rat brain extracts and fluorescence recovery after photobleaching assay indicated that NAP induced increased recruitment of human Tau3R to MTs under zinc intoxication, in comparison to Tau4R. Furthermore, we showed that NAP interaction with tubulin (MTs) was inhibited by obstruction of Tau-binding sites on MTs, confirming the requirement of Tau-MT interaction for NAP activity. The preferential interaction of NAP with Tau3R may explain clinical efficacy in mixed vs. Tau4R pathologies, and suggest effectiveness in Tau3R neurodevelopmental disorders.

## Introduction

Microtubules (MTs) are the major component of the neuronal cytoskeleton, and MT stability and organization play a critical regulatory role during axonal transport and synaptic transmission [1]. The MT-associated protein Tau is widely expressed in neurons and serves as a primary protein marker for axons [2, 3]. Tau promotes MT assembly and regulates MT dynamic instability, which is essential for establishing neuronal polarity, axonal elongation, and neural outgrowth [4]. Neurodegenerative disorders with Tau involvement are referred to as tauopathies [5]. The Tau protein consists of an N-terminus region projecting outward from the MTs and a C-terminus part directly interacting with the MTs through MT-binding domains [6]. Tau3R and 4R (containing either three or four MT-tubulin—binding repeats, respectively) are produced by alternative splicing around exon 10 of the Tau transcript [7]. The healthy human brain exhibits a 1/1 ratio of Tau3R/4R and deviation from this ratio are the pathological feature of several tauopathies [8]. Phosphorylation of Tau protein controls its binding to MT and is associated with Tau aggregation in neurodegenerative diseases [5, 9]. In general, Tau3R has been linked to neurodevelopment [7], while Tau4R with aging [10].

We have previously shown that the expression of activity-dependent neuroprotective protein (ADNP), a protein vital for brain formation [11, 12], is correlated with Tau3R expression [13] and Adnp+/- mice exhibit tauopathy features—significant increase in phosphorylated Tau, prevented by treatment of ADNP-derived peptide NAP (NAPVSIPQ) [14] as well as tangle-like structures. Our cell culture results have indicated that NAP enhances Tau-MT interaction in the face of zinc intoxication [15] and NAP protective activity requires Tau expression [16]. We have further revealed that NAP-Tau association is mediated by direct interaction of NAP and Tau with MT end-binding proteins (EBs) [15, 17].

Clinical trials identified the potential efficacy of NAP (davunetide, CP201) in enhancing short-term memory in amnestic mild cognitive impairment patients [18]. However, it was not found to be an effective (though, well tolerated) treatment for progressive supranuclear palsy (PSP) patients [19]. Because abnormal aggregation of Tau4R is a hallmark of PSP pathophysiology [20], the current study aimed to determine whether NAP had a different activity on either Tau3R or 4R. Our results now showed that NAP preferentially interacted with Tau3R protein from Sprague-Dawley rat brains and induced increased recruitment of human Tau3R to MTs under zinc toxic condition in comparison to Tau4R. Furthermore, we showed that NAP interaction with tubulin was inhibited by paclitaxel obstruction of Tau-binding sites on MTs, confirming the requirement of Tau-MT interaction for NAP activity.

## Results

### Tau from 60-day-old rat brain does not associate with NAP under conditions that Tau from newborn rat brain does

**Fig 1:**
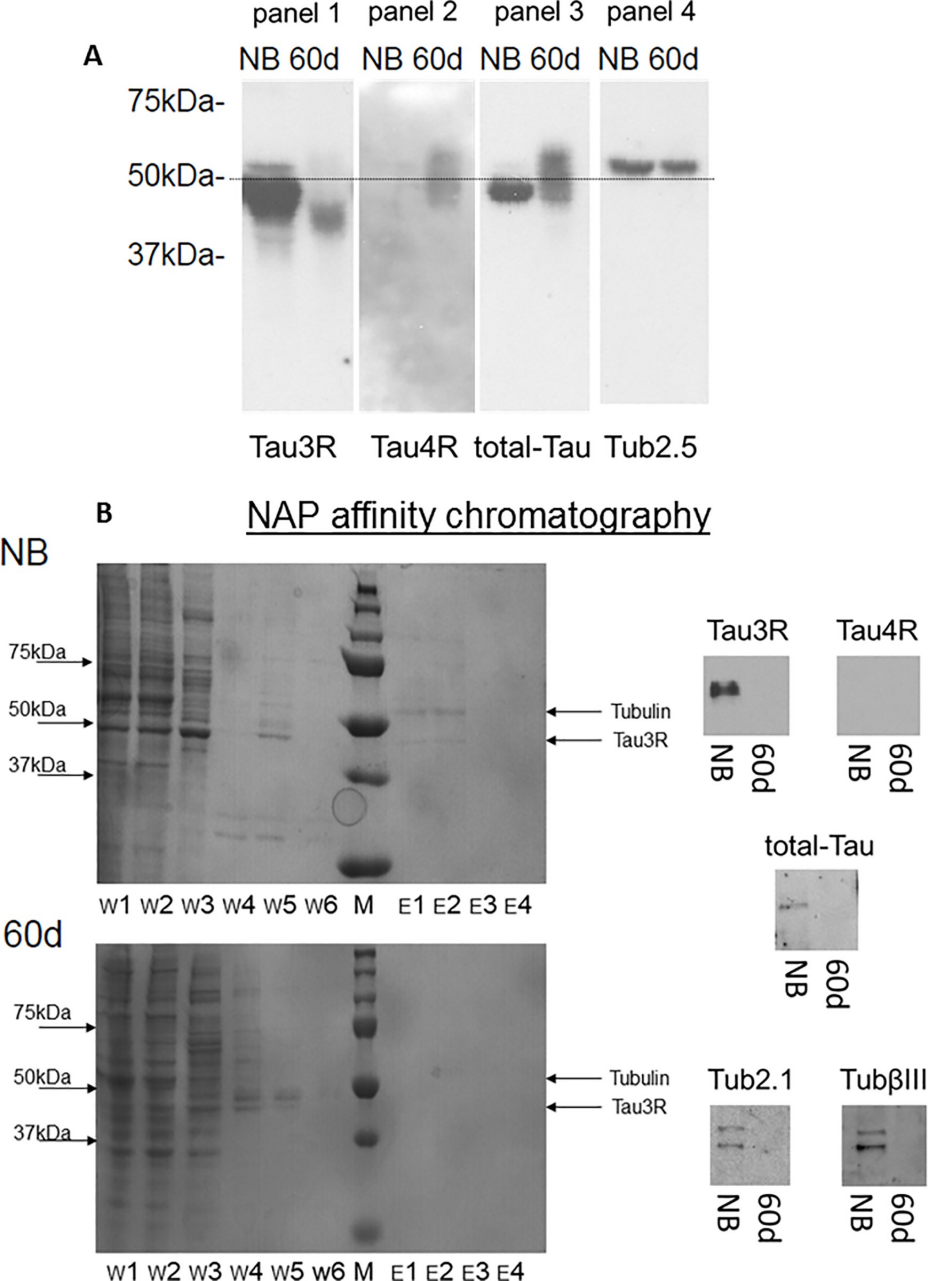
NAP preferentially associates with 3R-tau. **(A)** Western blot analysis of equal amounts of protein extracts from newborn (NB) rat brain cortex and 60-day-old (60d) rat brain cortex. Significantly larger amount of Tau3R was detected in the one-day-old cortical extract compared to the 60-day cortical extract, and Tau4R was recognized in the 60-day cortical extract only. **(B)** Bio-SafeTM Coomassie protein staining of the different fractions (F–flow-through, W—first wash, Wl—last wash, E1/2/3 –elution fractions by order) of the NAP affinity column fractions (left panel). Western blot analysis of elution fractions (E1) obtained by NAP-affinity chromatography with the same protein extracts of rat brain cortex (right panel). Tau3R, total-Tau, and tubulin were identified in the NAP-binding fraction of the newborn rat cortical brain extract, but essentially neither Tau nor tubulin was identified in the elution fraction of the 60-day-old cortical brain extract (three independent experiments). Please see S1 Fig for loading controls.

Different tubulin and Tau isotypes are expressed in the course of a rat brain development [10, 21]. Newborn-rats predominantly express the Tau3R isoform while adults predominantly express the Tau4R isoform [22]. Here, newborn and 60-day-old rat cerebral cortex extracts were analyzed by immunoblotting, and prevailing expression of Tau3R or 4R was observed in the newborn or 60-day-old cortex protein lysate, respectively, as expected (Fig 1A). However, some Tau3R immunoreactivity was observed in the 60-day-old rat cortex sample, but at a much lower intensity in comparison to Tau3R of the newborn brain protein extracts (Fig 1A, panel 1). This finding confirms the previously published data [23, 24] that detected trace amounts of Tau3R in the murine mature brain tissues. The 60-day-old Tau3R (in comparison to newborn Tau3R) exhibited lower molecular weight, which could represent different splice isoforms of the N-terminus of Tau3R. Notably, the number and intensity of the observed bands detecting Tau3R/4R and total-Tau cannot always be precisely compared because different antibodies exhibit different sensitivities and affinities to the various splice variants. Regardless, band intensity corresponding to a given protein within the panels (obtained from the same blot and probed with the same antibody, Fig 1A, panel 1 –Tau3R, panel 2 –Tau4R, panel 3 –total Tau, panel 4 –tubulin) could be compared. Thus, panel 1 revealed that the level of Tau3R in a mature rat cortex was indeed very low compared to the newborn brain (as detailed above). Panel 2 essentially did not detect Tau4R in the newborn rat cortex. However, panel 3 revealed that the levels of total-Tau in the newborn and mature rat cortex were similar, indicating that newborn Tau was mostly Tau3R, while 60-day-old Tau mostly consisted of Tau4R.

In a follow up experiment, newborn and 60-day-old rat cortex protein lysates were exposed to NAP affinity chromatography, and eluted proteins were separated by polyacrylamide gel electrophoresis (Commassie staining, Fig 1B, left panel) and analyzed by immunoblotting with Tau3R and 4R, total-Tau (identifying all Tau isoforms), tubulin Tub2.1 (identifying neuronal-enriched tubulin [25]) and TubβIII (identifying neuronal-specific tubulin [26]) antibodies. Immunoreactivity for all tested antibodies was detected in the acid eluted fraction from newborn rat brain extract (Fig 1B). However, no significant Tau or tubulin-like bands were observed in the eluted fractions from the mature rat extract under the current experimental conditions (Fig 1B). Notably, the absence of immunoreactivity in the mature rat brain extract eluates was corroborated by the absence of significant protein staining in the elution fractions (E1, E2), contrasting the protein staining and immune-detection in the newborn rat extracts (Fig 1B).

### NAP induces increased recruitment of human Tau3R to MTs under zinc toxic condition in comparison to Tau4R

**Fig 2:**
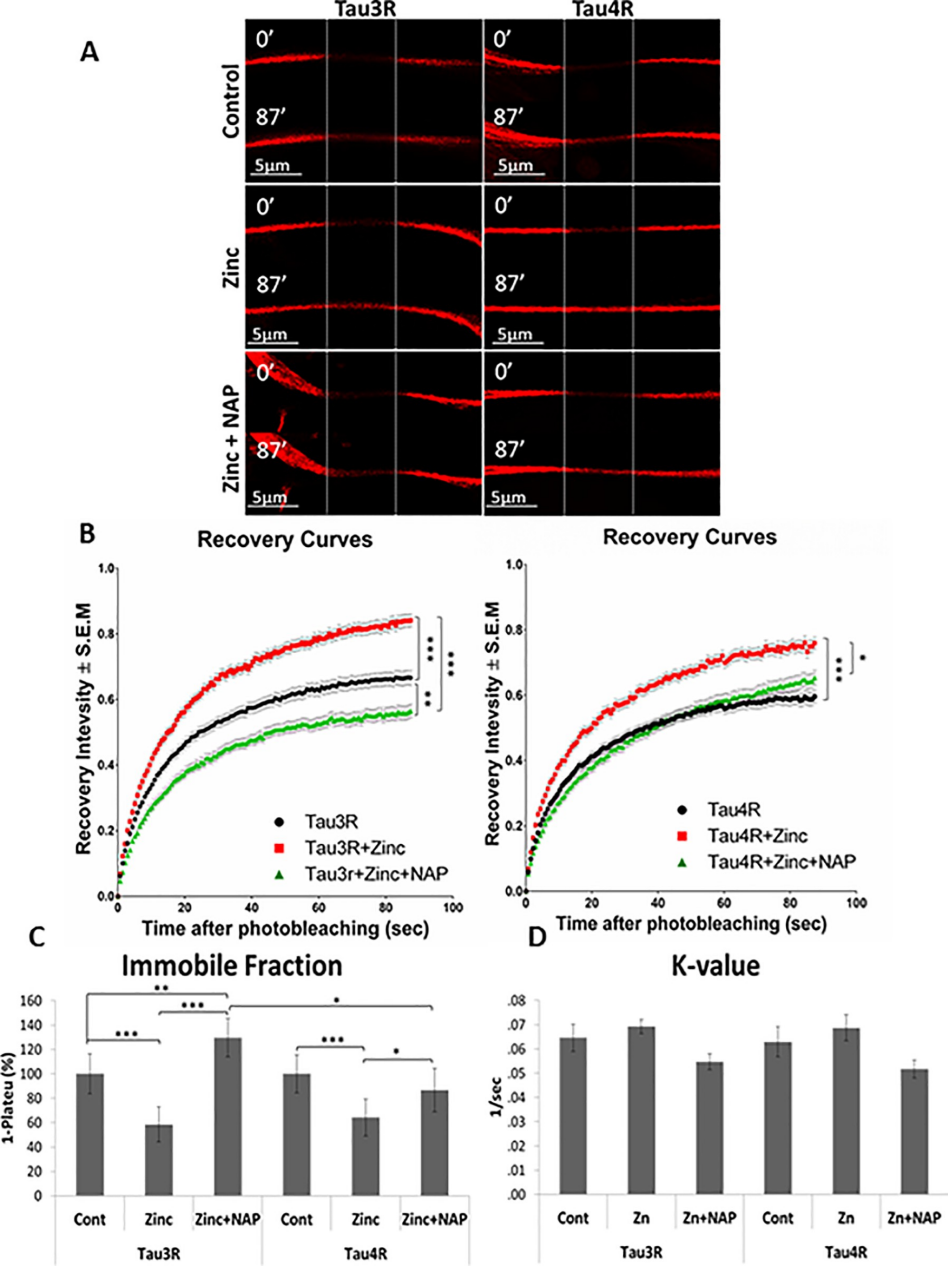
NAP induces increased recruitment of human Tau3R to MTs under zinc toxic condition in comparison to Tau4R. **(A)** Representative images of photo-bleaching and fluorescence recovery of mCherry-tagged Tau3R and 4R in differentiated N1E-115 cells treated with extracellular zinc (400μM, 2hrs) with or without NAP treatment (10^-12^M, 2hrs). N1E-115 cells expressing m-Cherry-Tau3R/4R without any treatment represented the control. **(B)** FRAP recovery curves of normalized data (see “Materials and Methods”). **(C)** The graph represents percentages (±SEM) of the fitted data (from three independent experiments) of immobile fractions relative to control– 100% (data were collected on 87 sec after photobleaching). Normalized FRAP data were fitted with one-exponential functions (GraphPad Prism 6), and statistical analysis was performed by Two Way ANOVA (SigmaPlot 11). Statistical significance is presented by *P<0.05, **P<0.01, *** P<0.001. Tau3R: Control n = 58, zinc n = 85, zinc + NAP n = 58; Tau4R: Control n = 56, zinc n = 47, zinc + NAP n = 60.

In order to test the effect of NAP on the interactions of different Tau isoforms with MTs, fluorescent recovery after photobleaching (FRAP) assay was performed (Fig 2). mCherry-tagged human Tau3R and 4R proteins (S2 Fig) were over-expressed in differentiated neuroblastoma N1E-115 cells and extracellular zinc (400μM, 1 hour) was used as a MT disruptor, inducer of Tau release from MTs [15]. In general, after photobleaching of the region of interest (ROI), the unrecovered portion of initial fluorescence intensity within a bleached area is referred to the immobile fraction of bleached mCherry-Tau proteins because it does not release binding sites on MTs for the entry of un-bleached mCherry-Tau molecules and thus does not allow fluorescence recovery. Therefore, the immobile mCherry-Tau fraction represents MT-bound Tau and reflects the MT-Tau interaction. Here, we observed that treatment with extracellular zinc increased fluorescence recovery 87sec after photobleaching of both mCherry-Tau3R and 4R molecules (Fig 2A). Analysis with one-phase exponential association showed a significant decrease of Tau3R and 4R immobile fractions (Fig 2B and 2C). NAP added together with zinc decreased fluorescence recovery (Fig 2A) and thus significantly enhanced the immobile fraction of Tau3R and 4R compared to treatment with zinc alone (Fig 2B and 2C). However, while the Tau4R immobile fraction was restored to untreated control level, the immobile fraction of Tau3R was further increased in comparison to control values and the difference between Tau3R and 4R immobile fractions was found statistically significant (Fig 2C). It should be noted that the rate constant (K-value) of mCherry-Tau fluorescence recovery (Fig 2D) although apparently different, was not significantly changed following different treatments. Regardless, quantitatively, NAP treatment produced a more potent impact on 3R-, rather than on 4R-Tau association with MTs.

### Tau interaction with MTs is required for NAP activity

**Fig 3:**
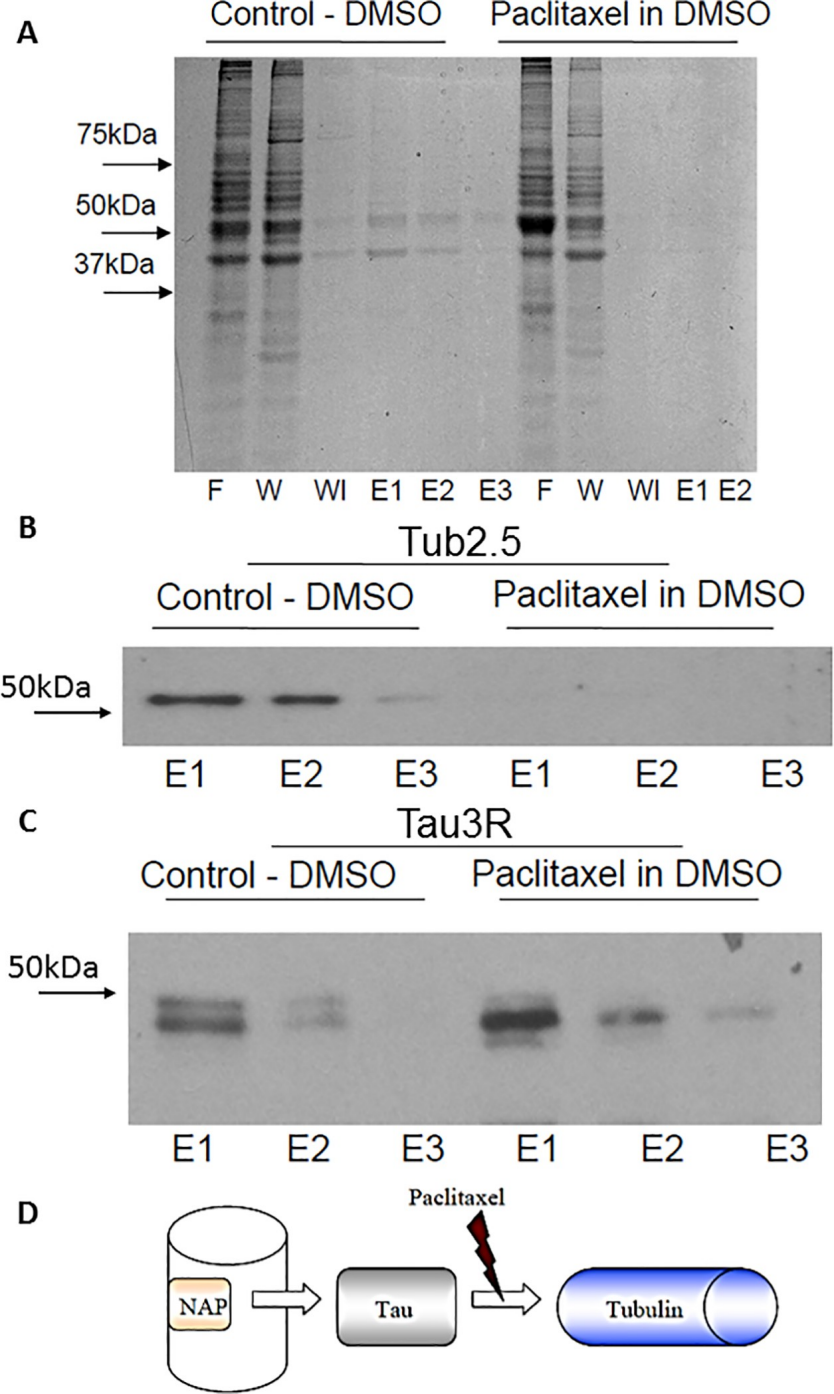
NAP interaction with tubulin, but not with Tau, is inhibited by paclitaxel. **(A)** Bio-SafeTM Coomassie protein staining of the different fractions (F–flow-through, W—first wash, Wl—last wash, E1/2/3 –elution fractions by order) obtained from NAP-affinity column loaded with protein extracts of newborn rat cerebral cortex with 4 mg paclitaxel, dissolved in 80μl DMSO, or equal volume of DMSO, alone. Almost no tubulin is evident in the elution fractions in the paclitaxel column, in contrast to the control one. (**B, C**) Western analysis of elution fractions obtained similarly as in panel (A). **(B)** Tubulin is not detected in the elution fractions of the pre-incubated paclitaxel column in comparison to the control column. **(C)** Tau3R is observed in the elution fractions of both the pre-incubated paclitaxel column and the control column. **(D)** Graphic depiction of the hypothesis that NAP, bound to an affinity column, interacts with tubulin throughout mediation of Tau.

Further, we aimed to test requirement of Tau-MT association for NAP interaction with tubulin/MTs. Because paclitaxel obstructs Tau-binding sites on tubulin [27] we incubated NAP-affinity columns with newborn cerebral cortical extracts in the presence of paclitaxel dissolved in DMSO or in the presence of DMSO alone (used as a control). Paclitaxel markedly decreased tubulin immunoreactivity in the acid-eluted fractions compared to the control (Fig 3A and 3B). Immunoreactivity of Tau3R appeared in both fractions–with and without paclitaxel exposure (Fig 3C). Careful assessment of the blots revealed several forms of Tau3R (please see discussion). As tubulin was washed away in the presence of paclitaxel, whereas Tau3R remained bound to the NAP column, we suggest that NAP interaction with tubulin required mediation of Tau (Fig 3D). To ascertain the specificity of NAP binding, an affinity control column with eight-amino-acids inactive peptide (VLGGGSALL) was prepared, as well. The peptide VLGGGSALL has previously shown no MT-related neuroprotective activity [28]. Affinity chromatography with the control peptide showed Tau3R presence in the loaded material, column flow-through, and column wash, but did not detect Tau3R in the acid elution fractions of both columns, neither in the absence nor in the presence of paclitaxel (S3 Fig). Also, tubulin was associated with VLGGGSALL regardless of paclitaxel presence, demonstrating nonspecific interaction (S3 Fig). The binding of tubulin (the major protein in brain extracts) included some non-specific association, as it also appeared in the control columns (S3 Fig.). Regardless, our further experiments with Tau3R (e.g. S3 Fig) showed specificity.

**Fig 4:**
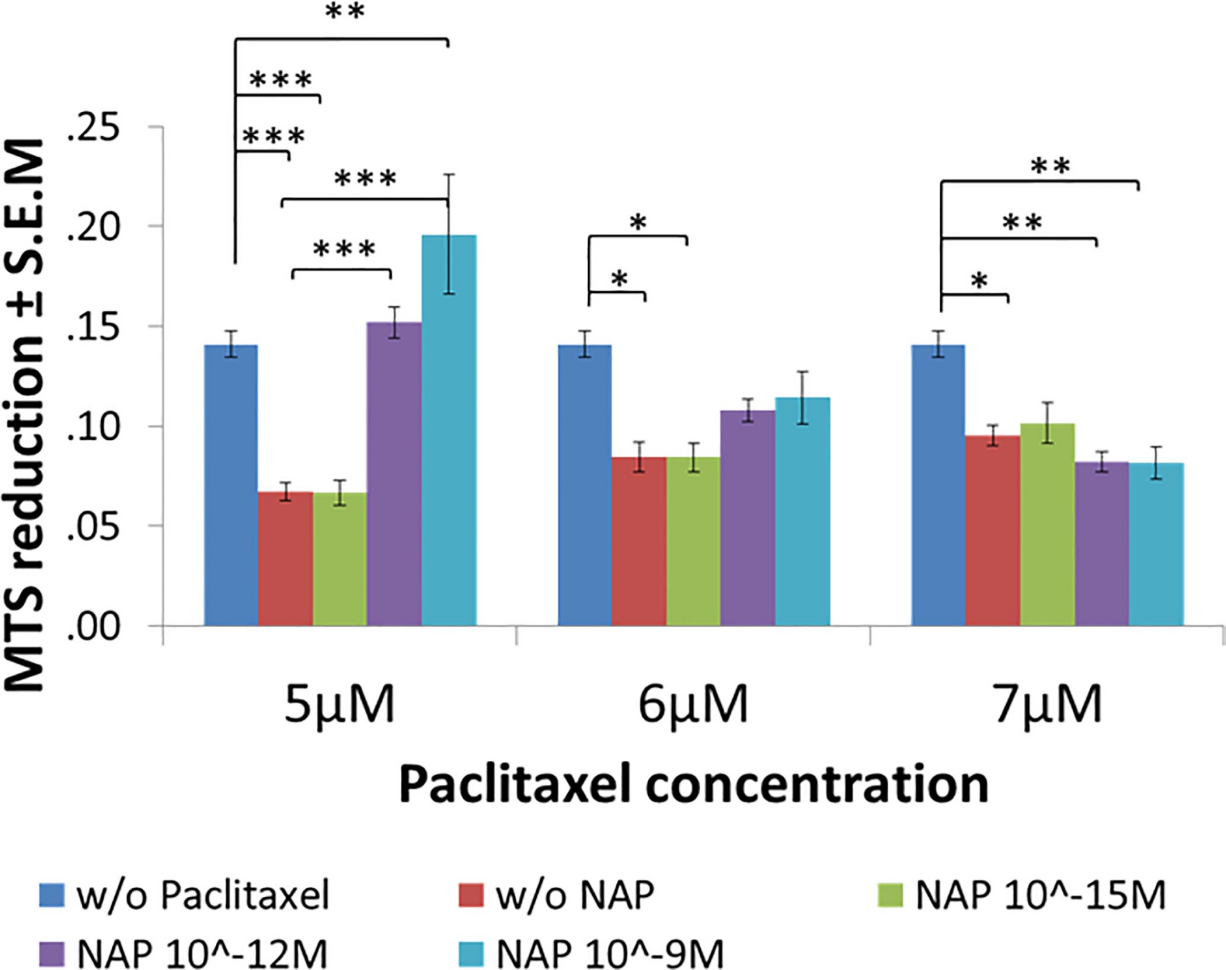
NAP protective activity is inhibited by paclitaxel. Cell viability test performed by the MTS assay (measures mitochondrial activity, see “Materials and Methods”). Differentiated N1E-115 cells are exposed to different concentrations of NAP (10^-15^M, 10^-12^M, 10^-9^M) and paclitaxel (5, 6 and 7 μM) for 2 hrs. Average of mitochondrial activity results (MTS reduction) are displayed in relation to the control (non-treated cell) value– 0.1411±0.02989. Statistical analysis of the data was performed using one-way ANOVA with Tukey post hoc test, n = 5. Statistical significance is presented relative to control as *P<0.05, **P<0.01, *** P<0.001; to “w/o NAP” (cells treated with paclitaxel, alone) as #P<0.05, ##P<0.01, ###P<0.001.

Then, we assessed the protective activity of NAP against increased concentrations of paclitaxel. For this purpose, differentiated N1E-115 cells were exposed to paclitaxel (5, 6 and 7 μM) with or without NAP (10^−15^, 10^−12^, 10^-9^M) for 4hrs. Cell viability, measured by mitochondrial activity, was significantly reduced following the 4hr-incubation period with paclitaxel. However, co-treatment with NAP (10^−12^ and 10^-9^M, but not 10^-15^M) protected against the lowest tested concentration of paclitaxel—5μM (Fig 4), but not against increased concentrations of paclitaxel– 6 and 7μM. These results suggest a requirement of direct Tau-MT interaction for NAP activity, confirming our previously published data that showed requirement of Tau expression for NAP protective capabilities [16].

### Phosphorylation patterns of human Tau4R affect NAP activity

**Fig 5:**
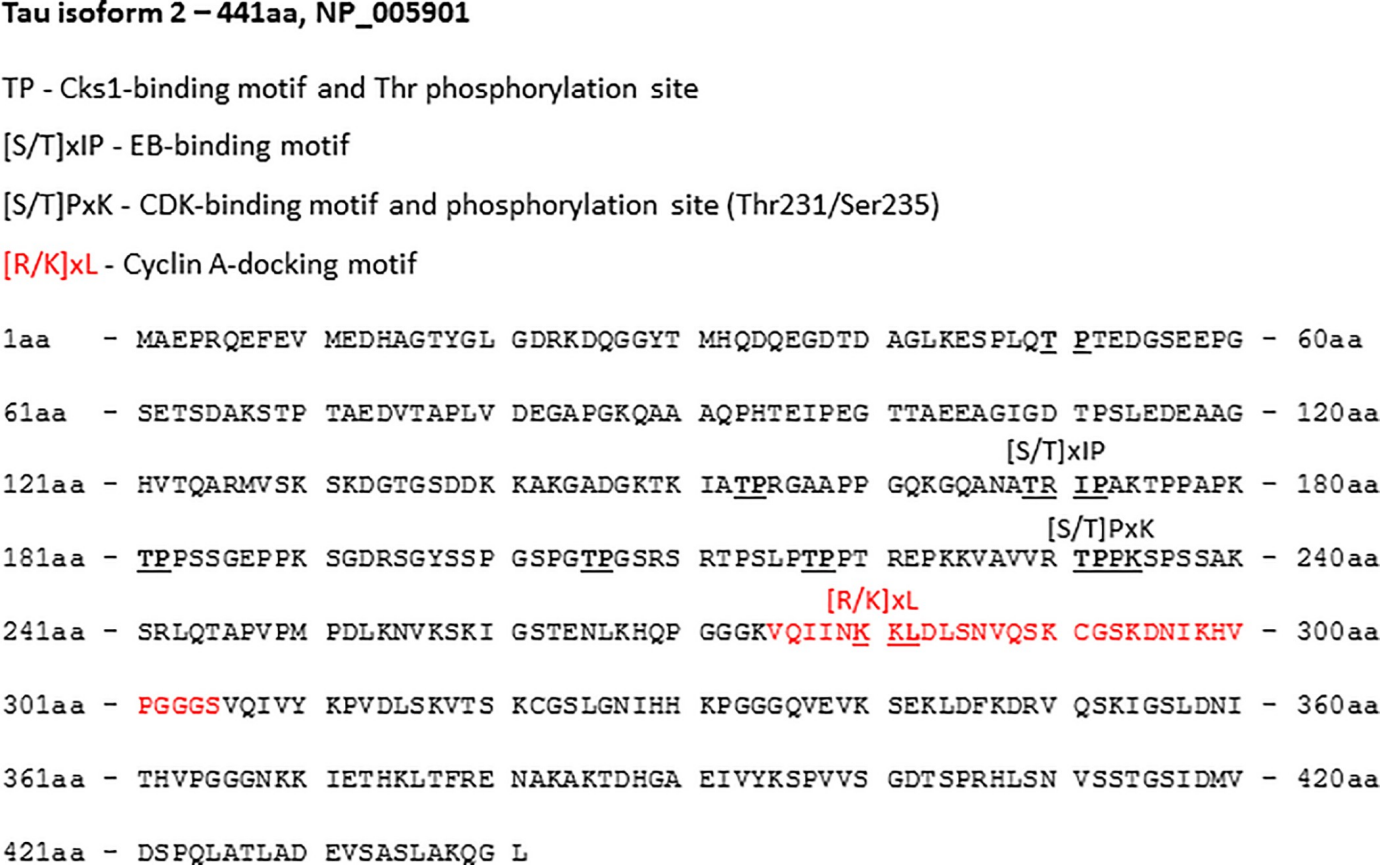
Amino-acid sequence of the Tau isoform 2 (NP_005901, 441aa) (Tau4R). The translated protein sequence of exon 10, which is spliced in Tau3R, is marked by red. Functional motifs, predicted by ELM analysis [30] (S1 Table and Table 1) are indicated.

NAP associates with Tau through its direct interaction with EB proteins [16, 17] and Tau-EB interaction may be affected by different phosphorylation states of Tau [29]. We now aimed to reveal the differences in the phosphorylation between human Tau3R and human Tau4R in the presence of NAP. ELM site prediction analysis [30] was performed to search the functional domains of Tau4R (MAPT) exon 10 (S1 Table and Fig 5, a sequence marked by red color), which is excised by alternative splicing leading to the production of Tau3R. The ELM prediction identified a cyclin A docking motif on the MAPT exon 10 coding region (S1 Table and Table 1) that recruits cyclin-dependent kinases (CDKs)–the well-known kinases stimulating Tau protein phosphorylation [31]. Additional ELM analysis of the whole Tau4R protein sequence revealed a CDK-binding motif and phosphorylation sites (Thr231/Ser235; Table 1, Fig 5) and multiple phosphorylation sites of the cyclin-dependent kinase subunit 1 (Cks1; Table 1, Fig 5), enhancing the specificity for CDK activity [32].

**Table 1:** ELM protein analysis. ELM predicted analysis [30] of Tau4R identified cyclin A docking motif, CDK-binding and phosphorylation site at threonine 231 and serine 235, and multiple phosphorylation sites of Cks1.

ELM Name	Matched Sequence	Position	ELM Description	Probability
DOC_CYCLIN_RxL_1	IINKKLDLS	277–285 [A] (the part of exon 10)	The classical cyclin docking motif pattern is mainly derived from peptides bound to Cyclin A	4.211e-03
MOD_CDK_SPxK_1	VVRTPPKS	228–235 [A]	Canonical version of the cyclin-dependent kinases (CDK) phosphorylation site	1.929e-03
DOC_CKS1_1	LQTPTE IATPRG PKTPPS PGTPGS LPTPPT VRTPPK	48–53 [A] 151–156 [A] 179–184 [A] 203–208 [A] 215–220 [A] 229–234 [A]	Phospho-dependent motif that mediates docking of CDK substrates and regulators to cyclin-CDK-bound Cks1	1.991e-03

**Fig 6:**
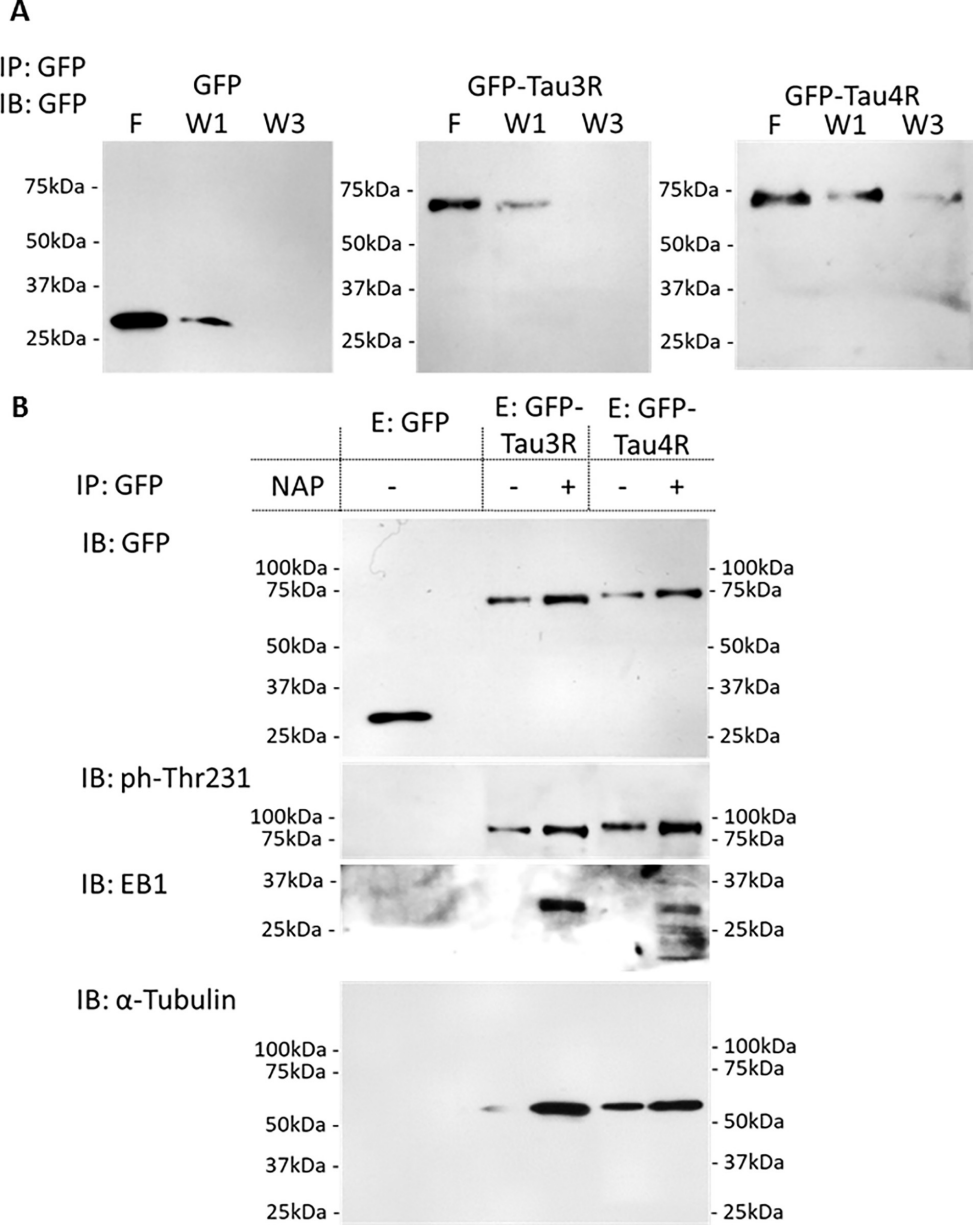
Increased phosphorylation of Tau4R reduces NAP effect on Tau-EB-tubulin interaction. Differentiated human neuroblastoma SH-SY5Y cells were transfected with expression plasmids encoding GFP-Tau3R or GFP-Tau4R. Cells expressing GFP only were used as negative controls. Immunoprecipitation (IP) was performed using GFP antibodies with and without NAP (see “Materials and Methods”). (A) Flow-through (F), first and third washes (W1 and W3) fractions were collected and analyzed by immunoblot with GFP antibody (IB: GFP). (B) Elution fractions (E) were collected and analyzed by immunoblotting (IB) with the appropriate antibodies, as listed on the figure.

Additionally, we aimed to test the ELM-predicted different phosphorylation profiles of Tau3R and 4R, and the effect of these on tubulin and EB interactions in the absence or presence of NAP. For this purpose, GFP-conjugated human Tau3R and 4R were over-expressed in differentiated human neuroblastoma SH-SY5Y cells and separately precipitated with GFP antibodies (Fig 6, IB: GFP panels). Further immunoblotting analysis with appropriate antibodies suggested increased phosphorylation on the Tau threonine 231 residue (Fig 6B, IB: ph-Thr231 panel) of Tau4R compared to Tau3R, in the absence of NAP addition. Incubation with NAP did not seem to robustly affect the ph-Thr231 of both Tau3R and 4R, in comparison to GFP-Tau ratios (Fig 5B, IB: GFP panel). Importantly, Tau-EB1 interaction was only observed in the presence of NAP, with an apparent increase EB1 association with Tau3R in comparison with Tau4R (Fig 6B, IB: EB1 panel). Notably, EB1 in the presence of Tau4R also showed some low molecular weight bands. Together, it is suggested that ph-Thr231 affects NAP-EB-Tau interactions.

Precipitated Tau4R in the absence of NAP showed increased interaction with tubulin in comparison to Tau3R (Fig 6B, IB: α-Tubulin panel), perhaps due to additional MT tubulin binding domain of Tau4R. Most importantly, NAP incubation strongly increased Tau3R-tubulin interaction, but only a minor increase in Tau4R-tubulin interaction was observed following NAP treatment (Fig 6B, IB: α-Tubulin panel, S5 Fig, overexposure). This confirmed the results above suggesting the preferential interaction of NAP with Tau3R in comparison with Tau4R. The use of human Tau species in human cells suggest applicability to the human conditions.

## Discussion

As dynamic tracks for motor proteins, MTs are involved in axonal transport and synaptic transmission. We have previously shown that NAP provides neuroprotection [28, 33] and neurotrophic activities [34] through interaction with MTs [15], rescues impaired axonal transport [35–37], regulates dendritic spines and enhances memory [38]. NAP also protects against the accumulation of pathologically modified, hyperphosphorylated Tau [14, 39–41]. We have previously suggested that the mechanism of NAP protective activity on MT-mediated cellular processes is through the involvement of Tau and MT end-binding proteins (EBs) [16, 35]. Specifically, NAP contains an ADNP association site (SIP) a signature motif for direct interaction with the EB1 and the EB3 proteins [35], which in turn bind to MTs [42] and Tau [17]. Furthermore, Tau has been identified as a regulator of EB’s action, and localization on MTs in developing neuronal cells [17] and NAP increases Tau-EB1/3 association [16]. Tau is important for the establishment of MT dynamic instability and axonal transport, while EB1 is more prevalent in neuronal axons [43] and EB3 in dendritic spines [44]. Formerly, it has been shown that expression of Tau and EB1/3 proteins are required for NAP-dependent neuronal survival [16, 35]. Here, we added details to the understanding of the molecular mechanism underlying the MT-related activity of NAP. Our current experiments showed that NAP preferentially interacted with rodent Tau3R (affinity chromatography) and induced enhanced recruitment of human Tau3R to MTs under zinc toxic condition in comparison to Tau4R (FRAP). Furthermore, we demonstrated that paclitaxel-disturbed Tau-tubulin interaction prevented NAP association with tubulin/MTs and inhibited NAP protective activity, suggesting the requirement of not only the expression of Tau, but also Tau-tubulin direct association for sufficient action of NAP.

**Fig 7:**
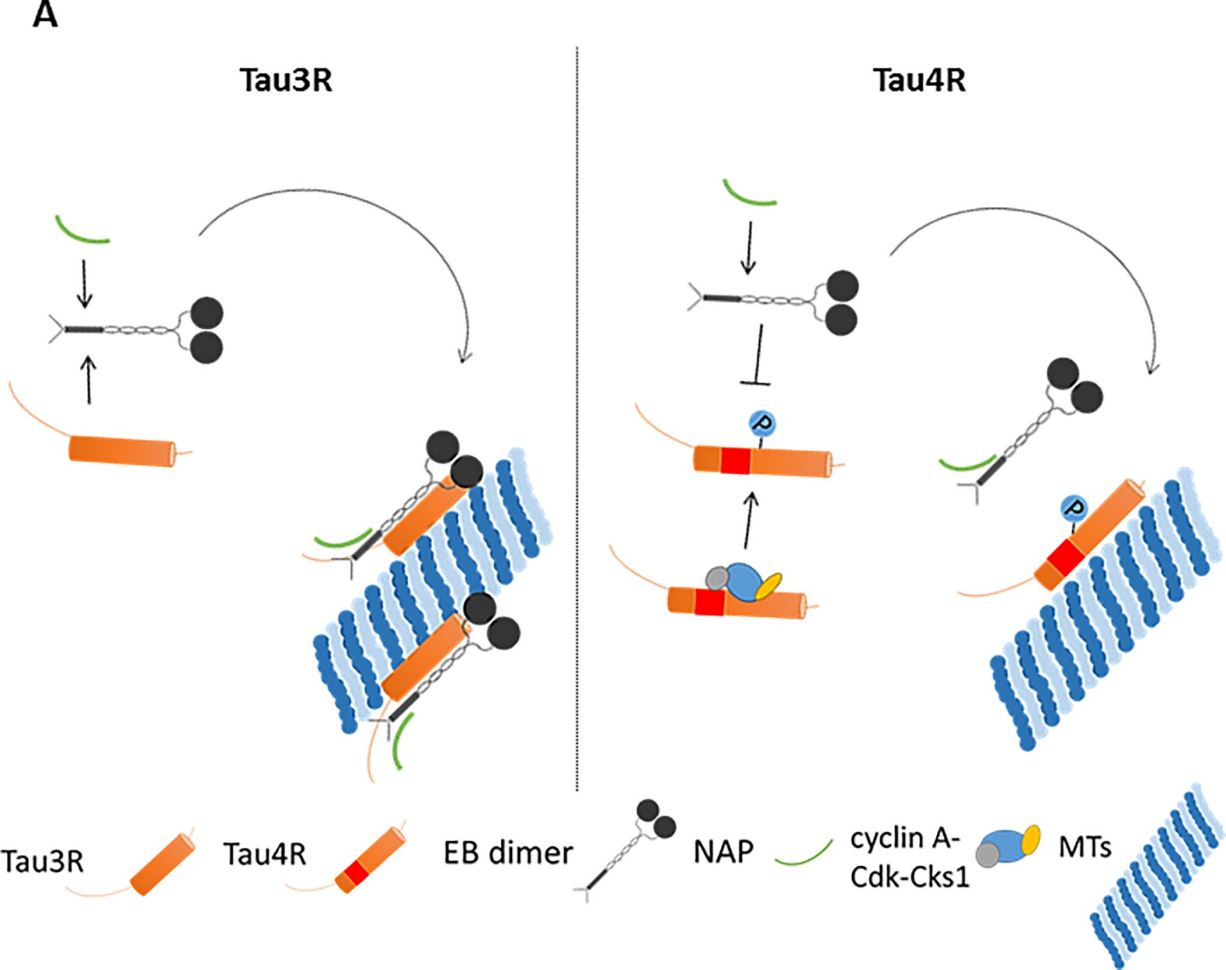
Suggested explanation for the preference of NAP binding to Tau3R over Tau4R. Graphic depiction of our hypothesis suggesting the preference of NAP to interact with Tau3R based on the findings of the ELM prediction analysis and experimental results presented in Fig 6. The second MT-binding repeat of Tau4R (spliced in Tau3R) includes cyclin A-docking motif and thus may enable Tau binding to cyclin A, essential for the activation of Cdk1/2 [31]. Cks1 may also associate with Cdk and cyclin A to form more efficient cyclin A-Cdk-Csk1 phosphorylation complex. Tau4R phosphorylation by Cdk1/2 differs from Cdk5 (a conventional Tau kinase) [46] and may disturb/attenuate Tau-EB interaction, which has been previously indicated as a crucial for NAP interaction with MTs [16, 35]. The EB dimer structure was constructed according to a published review [42].

NAP affinity chromatography with paclitaxel increased NAP-Tau interaction that resulted in an additional Tau immunoreactive band appearance in comparison to treatment conditions without paclitaxel (Fig 3C). A trivial explanation to the additional faint tau band in the eluate of the affinity column could be related to the increased protein content eluted in the presence of paclitaxel. However, the detection of an additional splice variant affecting the N-terminal of the protein, or a different phosphorylation state could not be ruled out. The only difference between the two Tau isoforms (3R and 4R) is the presence of the exon 10 coding sequence comprising an extra MT-binding repeat in Tau4R (Fig 5, red sequence) which is excluded during alternative splicing in Tau3R [45]. ELM prediction analysis [30] of the whole Tau sequence identified cyclin A-docking motif within the translated sequence of exon 10 (Fig 5, S1 Table). Whereas cyclin-dependent kinase (Cdk) 5 is activated by non-cyclin proteins, Cdk1/2 requires direct association with cyclin A imposing an active conformation on the kinase [31]. Furthermore, six docking/phosphorylation sites of the cyclin-dependent kinase subunit 1 (Cks1) were identified by ELM prediction on Tau (Fig 5, Table 1). Cks1 association with the Cdk-cyclin complex increases the specificity and efficiency of Cdk substrate phosphorylation [32]. It has been reported that Cdk2 and Cdk5 provide different Tau phosphorylation profiles [46]. It was further reported that region-specific Tau phosphorylation might attenuate Tau-EB association [29]. Because NAP interacts with Tau through EB proteins, and Tau3R and 4R may present differences in the phosphorylation profiles, we speculated that observed attenuation of NAP-Tau4R interaction occurs due to some phosphate incorporation on Tau and ensuing decrease of EB protein association, as now shown for Thr231 in Figs 6 and 7. Furthermore, we have previously reported that NAP reduces Tau phosphorylation at Ser262 [36], Ser202/Thr205, and Thr231 [47] residues, but does not exhibit a significant impact on Tau phosphorylation level at Thr181 [19]. Intriguingly, Thr181 is on one of the predicted Tau binding/phosphorylation motifs of Cks1 modulating the activity of Cdk (Fig 5, Table 1).

As opposed to rodents, Tau3R is abundant alongside with Tau4R in the human adult brain. The ratio of Tau 3R:4R proteins is important, and changes in the ratio are observed in tauopathies [48]. NAP does not interact with MT proteins from various cancer cell lines or fibroblasts [33], which may not express any MT-associated proteins with properties that are similar to Tau3R, and NAP does not affect cell division [49]. Furthermore, NAP does not protect cells from fibroblast origin unless those cells are transfected with Tau3R-expressing plasmid [16]. However, NAP protects MT organization in mature neurons and glia [28, 33, 50]. We have previously shown that by promoting the interactions of Tau and EBs with MTs, NAP protects MTs against degradation and concurrently enhances MT dynamics [16]. *In vivo*, chronic NAP treatment reduces excess Tau accumulation and pathological hyperphosphorylation [14, 40, 47]. Our studies explain, in part, the efficacy of NAP (davuntide, CP201) in enhancing cognitive functions in mild cognitive impairment patients [18], while showing no efficacy (although high safety) in the 4R tauopathy progressive supranuclear palsy (PSP) [19]. Since tauopathy underlies a verity of neurodegenerative conditions, our current findings may pave the path for treatment by peptide drugs that have an impact on tubulin-Tau interaction and specific neurofibrillary tangle populations [51]. As neurodevelopment has been linked to Tau3R [7, 10], our results pave the path to the development of NAP (davunetide, CP201) for ADNP deficiencies associated with neurodevelopment, for example, the autism-like ADNP syndrome, resulting from *de novo* truncating mutations in ADNP (32).

## Materials and methods

### Ethical statement

Animal studies were approved by the Institutional Animal Care and Use Committee (IACUC) of Tel Aviv University. Approval number: M-06-008. Animals were anesthetized by Ketamine (20mg per 10gr of animal weight)/Xylazine (0.2mg per 10gr of animal weight) injection and brains were extracted following dislocation.

### Brain extract preparation

Protein lysate was prepared from either one- or sixty-day-old Sprague-Dawley rat cerebral cortex in a lysis buffer containing: 150 mM NaCl, 1 mM EDTA, 50 mM Tris-HCl, pH 4.5 (or 7.5 as indicated), 0.1% Triton X-100, 1% Nonidet P-40, and a protease inhibitor cocktail (Roche Diagnostics, Mannheim, Germany). DNA was fragmented by sonication. Cell debris was discarded following 20 minutes of centrifugation at 30,000g at 4°C, as described previously [28].

### NAP affinity chromatography

Affinity columns contained extended NAP (**CKKKGG**NAPVSIPQ, the linker peptide is in bold). Peptides were purchased from Genemed Synthesis, Inc., San Antonio, TX, USA or synthesized as before [52]. Columns and peptide binding were prepared using Sulfolink coupling gel (Pierce, Rockford, IL, USA) according to the manufacturer’s instructions as before [28]. 2 ml Sulfolink coupling gel was loaded onto Poly-Prep Chromatography Columns (Bio-Rad, Hercules, CA, USA) and coupled with 2mg/ml peptide. Coupling was ascertained by free peptide measurements.

In order to determine the binding specificity of Tau and tubulin to NAP, multiple experiments were performed under stringent comparable experimental conditions as follows:

1] Proteins from either newborn or sixty-day-old rat cerebral cortical extracts (2mg protein/ml, total volume 2 ml) were loaded onto the NAP-affinity columns at pH 7.5 and incubated for 16 hours at 4°C, columns were washed with phosphate buffered saline (PBS, 20–25 ml) until all unbound protein had eluted as confirmed by the Bradford protein assay (Bradford, BioRad, Hercules, CA, USA). The bound protein was then eluted with 0.1 M glycine pH 2.6.

2] Proteins from one-day-old rat (expressing Tau3R, solely) cerebral cortical extracts were loaded onto the NAP column with 4 mg paclitaxel (Haorui Pharma-Chem Inc., New-Jersey, USA) dissolved in 80μl dimethyl sulfoxide (DMSO, Sigma, Rehovot, Israel). A control NAP column was treated similarly with 80μl DMSO but without paclitaxel. The columns were incubated with brain extract, washed as described above, and eluted with glycine 0.1 M pH 2.6.

3] The control column contained an inactive peptide **CKKKGG**VLGGGSALL (the linker peptide is in bold) described in supplemental materials and methods (S1 File and S3 Fig).

### SDS-PAGE and western blot analysis

The flow-through, wash fractions and elution fractions were separated by 10% or 12% SDS polyacrylamide gel electrophoresis (SDS-PAGE) followed by protein staining using Bio-SafeTM Coomassie (Bio-Rad, Hercules, CA, USA) according to manufacturer’s instructions or transferred to nitrocellulose membranes (Schleicher and Schull, Dassel, Germany) for western blot analysis. In comparative experiments, run in parallel, the same amounts were loaded on the gels, for each parallel fraction. Non-specific sites on the nitrocellulose membranes used for western analysis were blocked in a blocking solution (10 mM Tris pH 8, 150 mM NaCl, and 0.05% Tween 20 [TBST]) supplemented with 5% non-fat dried milk (1 hour, at room temperature). The Protein complexes were visualized by SuperSignal West Pico Chemiluminescent Substrate (Pierce, Rockford, IL, USA).and exposed on Fuji Film Medical X-ray film (Fuji Corporation, Tokyo, Japan).

### Antibodies

Total-Tau—mouse monoclonal antibody Tau5 (antibody recognizing all Tau forms) was obtained from MBL International Corporation (Woburn, MA, USA). Tau3R and Tau4R - mouse monoclonal anti-Tau RD3 (3-repeat isoform) and mouse monoclonal anti-Tau RD4 (4-repeat isoform) were obtained from Millipore Corporation (Billerica, MA, USA). Tub2.1 and Tub2.5—mouse monoclonal tubulin antibodies maintained and kindly provided by Professor Colin J. Barnstable, and were used as before [25]. TubβIII—mouse monoclonal antibody β tubulin isotype III was obtained from Sigma-Aldrich (St. Louis, MO, USA). α-Tubulin—monoclonal anti-α-Tubulin (mouse IgG1 isotype) (T6199, Sigma, Rehovot, Israel) recognizes an epitope located at the C-terminal end of the α-tubulin isoform. Ph-Thr231—mouse monoclonal anti-phospho-Tau at threonine 231 residue (dilution 1:500; clone AT180, Thermo Fisher Scientific, Inc., Waltham, MA, USA). EB1 –rat monoclonal anti-MAPRE1 (clone KT51, Abcam, Berlin, Germany). GFP–mouse monoclonal anti-GFP antibody (dilution 1:2000; sc-9996, Santa Cruz Biotechnology, Inc.; Dallas, Texas, USA). Secondary antibodies were goat anti-mouse-horseradish peroxidase—HRP (Jackson ImmunoResearch, West Grove, PA, USA). All antibodies were used at the dilution of 1:1000, except when otherwise indicated.

### Plasmid construction

DNA inserts carrying Tau3R and 4R were obtained from human Tau3R and 4R cDNA containing plasmids (a kind gift of Professor M. Goedert, MRC Laboratory of Molecular Biology, Cambridge, UK) and then cloned into the backbone of pEGFP-C1 or the newly constructed pmCherry-C1 plasmid. For more details, see supplemental materials and methods (S1 File and S1 Fig).

### Cell culture and treatments

Mouse neuroblastoma N1E-115 cells (ATCC, Bethesda, MD; passage numbers from 10 to 13) were maintained in Dulbecco’s modified Eagle’s medium (DMEM), 10% fetal bovine serum (FBS), 2 mM glutamine and 100 U/ml penicillin, 100 mg/ml streptomycin (Biological Industries, Beit Haemek, Israel). Human neuroblastoma SH-SYS5 cells (ECACC, Public Health England, Porton Down, Salisbury, UK; passage numbers from 14 to 16) were maintained in Ham's F12: minimum essential media (MEM) Eagle (1:1), 2mM Glutamine, 1% non-essential amino acids, 15% fetal bovine serum (FBS) and 100 U/ml penicillin, 100 mg/ml streptomycin (Biological Industries, Beit Haemek, Israel). The cells were incubated in 95% air/5% CO_2_ in a humidified incubator at 37°C. N1E-115 cells were plated on 35mm dishes (81156, 60 μ-Dish, Ibidi, Martinsried, Germany) at a concentration of 25*10^4^ cells/dish and then were differentiated with reduced FBS (2%) and DMSO (1.25%) containing medium during five days before transfection and seven days before the experiment. On the day of the experiment, differentiated N1E-115 cells were treated for 1 hrs with zinc chloride (ZnCl_2_; final concentration, 400 μM, Sigma, Rehovot, Israel) with or without NAP (10^−12^M). Cultured SH-SY5Y cells were plated in 10cm dishes at a concentration of 0.5*10^6^/dish and differentiated with retinoic acid at a concentration of 10 μM during seven days.

### Transfection of over-expression plasmids and Fluorescence recovery after photobleaching (FRAP)

5-day differentiated N1E-115 cells were transfected with a 1μg pm Cherry-C1-Tau3R/4R plasmid. 48 hrs after transfection, cultured N1E-115 cells were incubated at 37°C with a 5% CO_2_/95% air mixture in a thermostatic chamber placed on the stage of a Leica TCS SP5 confocal microscope [objective 100x (PL Apo) oil immersion, NA 1.4]. An ROI (region of interest) for photo-bleaching was drawn in the proximal cell branches. mCherry-Tau3R/4R was bleached with a 587nm argon laser, and fluorescence recovery was at 610-650nm. Immediately after bleaching, 80 images were collected every 0.74s. Fluorescence signals were quantified with ImageJ (NIH), obtained data were normalized with easyFRAP43, and FRAP recovery curves were fitted by a one-phase exponential association function using GraphPad Prism 6 (GraphPad Software, Inc., La Jolla, CA). Samples with R2<0.9 were excluded.

### Cell viability assay

7-day differentiated N1E-115 cells were treated with different concentrations of NAP (10^-15^M, 10^-12^M and 10^-9^M) and paclitaxel (5, 6 and 7μM diluted in DMSO) for 2 hours. Treatments with 5, 6 and 7μM of DMSO alone were used as controls. Cell viability was measured using the MTS assay (CellTiter 96 AQueous Non-Radioactive Cell Proliferation Assay; Promega, Madison, WI, USA), which was performed according to the manufacturer's instructions and read in an ELISA plate reader at 490nm.

### Transfection plasmids and immunoprecipitation assay (IP)

Differentiated SH-SY5Y cells were transfected with 8μg GFP-C1-Tau3R/4R plasmid or control GFP plasmid. 48 hrs after transfection, proteins were extracted with lysis buffer (Pierce, Rockford, IL) with added protease inhibitor (11255500, Roche, Mannheim, Germany). Immunoprecipitation (IP) was performed with GFP-Trap A beads according to the provided protocol (ChromoTek Inc., Planegg-Martinsried, Germany). 2.3μg of NAP, diluted into lysis buffer (NAP 2.3μg/sample), or the equal volume of lysis buffer w/o NAP were added to lysates of the transfected SH-SY5Y cells. Protein lysate with or without NAP was added to equilibrated GFP-Trap A beads and incubated 2 hrs at +4°C under constant mixing. Flow-through, wash 1 and 3, and elution fractions were collected and analyzed by immunoblotting with the appropriate antibodies.

### Statistical analysis

Data are presented as the mean ± SEM from 3 independent experiments. Statistical analysis of the data was performed by using one-way ANOVA test (followed by the Tukey post hoc test) by the IBM SPSS Statistics software version 23. Two-way ANOVA was implemented when needed. * P<0.05, ** P<0.01, *** P<0.001.

## Supporting information

S1 File Supplemental materials and methods. Plasmid construction and Affinity chromatography with eight-amino-acids inactive peptide (VLGGGSALL).
(DOCX)

S1 Fig Column loading controls. Coomassie staining. M–protein ladder; 1, 2 –Total rat brain extract loading controls.
(PDF)

S2 Fig Plasmid maps. pmCherry-C1 and pEGFP-C1 vectors (A), human Tau3R **(B)** and 4R **(C)** expressing plasmids based on pmCherry-C1 and and pEGFP-C1 vectors. The plasmid maps were constructed with Benchling platform (www.benchling.com).
(PDF)

S3 Fig VLGGGSALL does not interact with Tau3R. The gel lanes contain the protein loaded (load) flow-through (FT) PBS wash, pH7.5 (W1-30) and acid elutes (E1-E4) from the columns linked to eight-amino-acid inactive peptide VLGGGCALL P (has previously shown no microtubule-related neuroprotective activity [28]) that were incubated with brain extracts and DMSO in the absence and presence of paclitaxel. Western blotting analysis with anti Tau RD3 detected Tau3R presence in the loaded material, column flow-through and column wash, but did not detect Tau3R in the acid elution fractions of both the columns. In contrast, tubulin antibodies—Tub2.5 identified tubulin-like bands also in the elution fractions with no apparent influence of paclitaxel treatment.
(PDF)

S4 Fig Two-way ANOVA statistical analysis. Examination of the effect of the two factors (Paclitaxel and NAP) showed that NAP had a significant effect only for the lower paclitaxel dose (D = 5). The indicated p-value is based on one-way ANOVA for this group; figure was generated using R.
P-values of two-way ANOVA: paclitacel—0.00257; NAP—0.01093; paclitaxel:NAP interaction—3.58e-10.
(PDF)

S5 Fig Immunoblotting with tubulin antibody–overexposed cellulose membrane presented in the Fig 6B, panel IB. α-Tubulin. Differentiated human neuroblastoma SH-SY5Y cells were over-expressed with GFP-Tau3R or GFP-Tau4R. Cells with GFP expression were used as negative control. Immunoprecipitation (IP) of GFP, GFP-Tau3R and GFP-Tau4R in the presence and absence of NAP was done with GFP antibody. Elution fractions (E) analyzed by immunoblotting (IB) with tubulin antibody.
(PDF)

S1 Table ELM prediction analysis of Tau (NP_005901) exon 10 translation sequence. ELM analysis [30] predicted functional motifs of the translation sequence of spliced exon 10 (VQIINKKLDLSNVQSKCGSKDNIKHVPGGGS) of Tau isoform 2 (NP_005901). DOC_CYCLIN_RxL_1 motif appeared only once in full Tau sequence.
(DOCX)

S1 Dataset Minimal dataset is available in a supplemental file named: Raw_data. (XLSX)

S1 ARRIVE Checklist NC3Rs ARRIVE Guidelines Checklist (fillable) was completed as required. (PDF)
