# Supplementary material for: NAP (davunetide) preferential interaction with dynamic 3-repeat Tau explains differential protection in selected tauopathies
Source: PLoS One. 2019 Mar 13;14(3):e0213666. doi: 10.1371/journal.pone.0213666 (PMC6415897; doi:10.1371/journal.pone.0213666)
Supplement: S1 File — Plasmid construction and Affinity chromatography with eight-amino-acids inactive peptide (VLGGGSALL). (DOCX) [file pone.0213666.s001.docx]

**Supplemental Materials and Methods**

ADNP-derived peptide NAP preferentially interacts with 3-repeat Tau isoform and modulates Tau-tubulin association:

Elaborate molecular mechanism of NAP neuroprotective activity.

Yanina Ivashko-Pachima^1^, Maya Maor^1^ and Illana Gozes^1^*

^1^Department of Human Molecular Genetics and Biochemistry, Sackler Faculty of Medicine, Sagol School of Neuroscience and Adams Super Center for Brain Studies, Tel Aviv University, Tel Aviv, Israel.

*Corresponding author

Illana Gozes, Ph.D., Professor of Clinical Biochemistry

E-mail: igozes@post.tau.ac.il

**Plasmid construction**

The following backbone plasmids were used for cloning: enhanced green fluorescent protein (EGFP) DNA-coding sequence from pEGFP-C1 and newly formed pmCherry-C1. EGFP-coding sequence was substituted by DNA-coding sequence of mCherry fluorescent protein to obtain pmCherry-C1 vector (Supplemental Fig. S2A). Cloning was done by cleavage digesting with appropriate restriction enzymes (AgeI R0552 and BglII R0144, New England Biolabs® Inc., Ipswich, MA, USA). Human Tau3R and 4R DNA inserts, overlapped with appropriate restriction enzymes sites (EcoRI R0101 and BamHI R0136, New England Biolabs® Inc., Ipswich, MA, USA), were obtained from human Tau3R and Tau4R cDNA containing plasmids (a kind gift of Professor M. Goedert, MRC Laboratory of Molecular Biology, Cambridge, UK) by PCR (Q5 high-fidelity DNA polymerase, M0491, New England Biolabs® Inc., Ipswich, MA, USA) and cloned into pEGFP-C1 and pmCherry-C1 vectors (S2B and C Figs). After cloning, the insert regions were sequenced to validate the in-frame insertion and desired DNA sequence.

**Affinity chromatography with eight-amino-acids inactive peptide (VLGGGSALL)**

To ascertain specificity of NAP binding an affinity control column was prepared in parallel to a NAP affinity column. The control column contained an inactive peptide **CKKKGG**VLGGGSALL (the linker peptide is in bold). Equal amounts of cerebral cortical extracts (2 mg/ml) from newborn rats were applied onto columns. 4mg paclitaxel was added to one of the columns together with the brain extract, while the other column served as a control in the presence of DMSO (as described for NAP affinity columns). The columns were incubated and washed as described for NAP affinity columns, and eluted with glycine 0.1 M pH 2.6 (S3 Fig).
