# Supplementary figures and images for: NAP (davunetide) preferential interaction with dynamic 3-repeat Tau explains differential protection in selected tauopathies
Source: PLoS One. 2019 Mar 13;14(3):e0213666. doi: 10.1371/journal.pone.0213666 (PMC6415897; doi:10.1371/journal.pone.0213666)

**Figure S1**

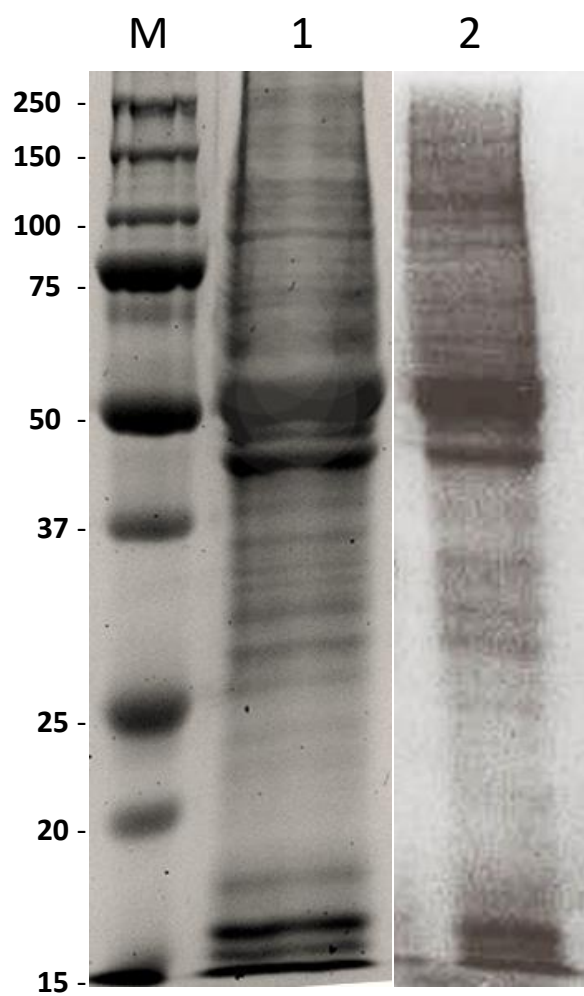

Supplement: S1 Fig — Coomassie staining. M–protein ladder; 1, 2 –Total rat brain extract loading controls. (PDF) [file pone.0213666.s002.pdf]

(A)

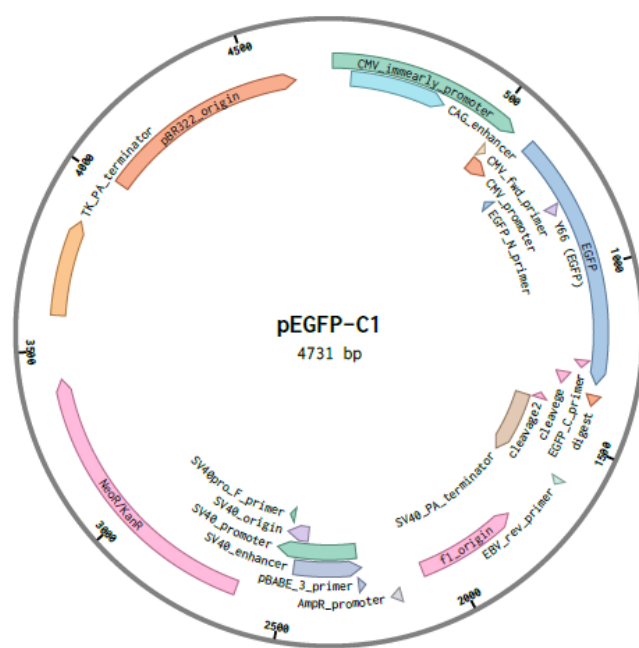

(B)

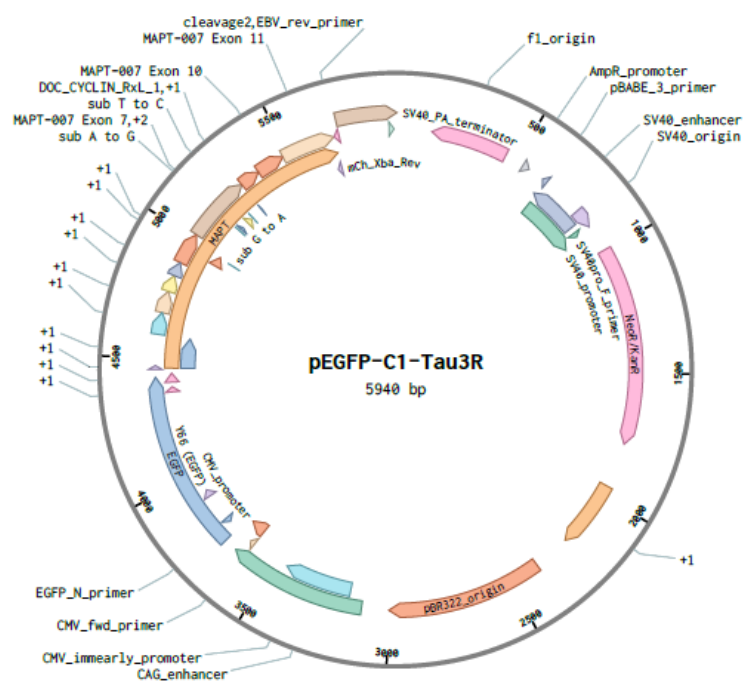

(C)

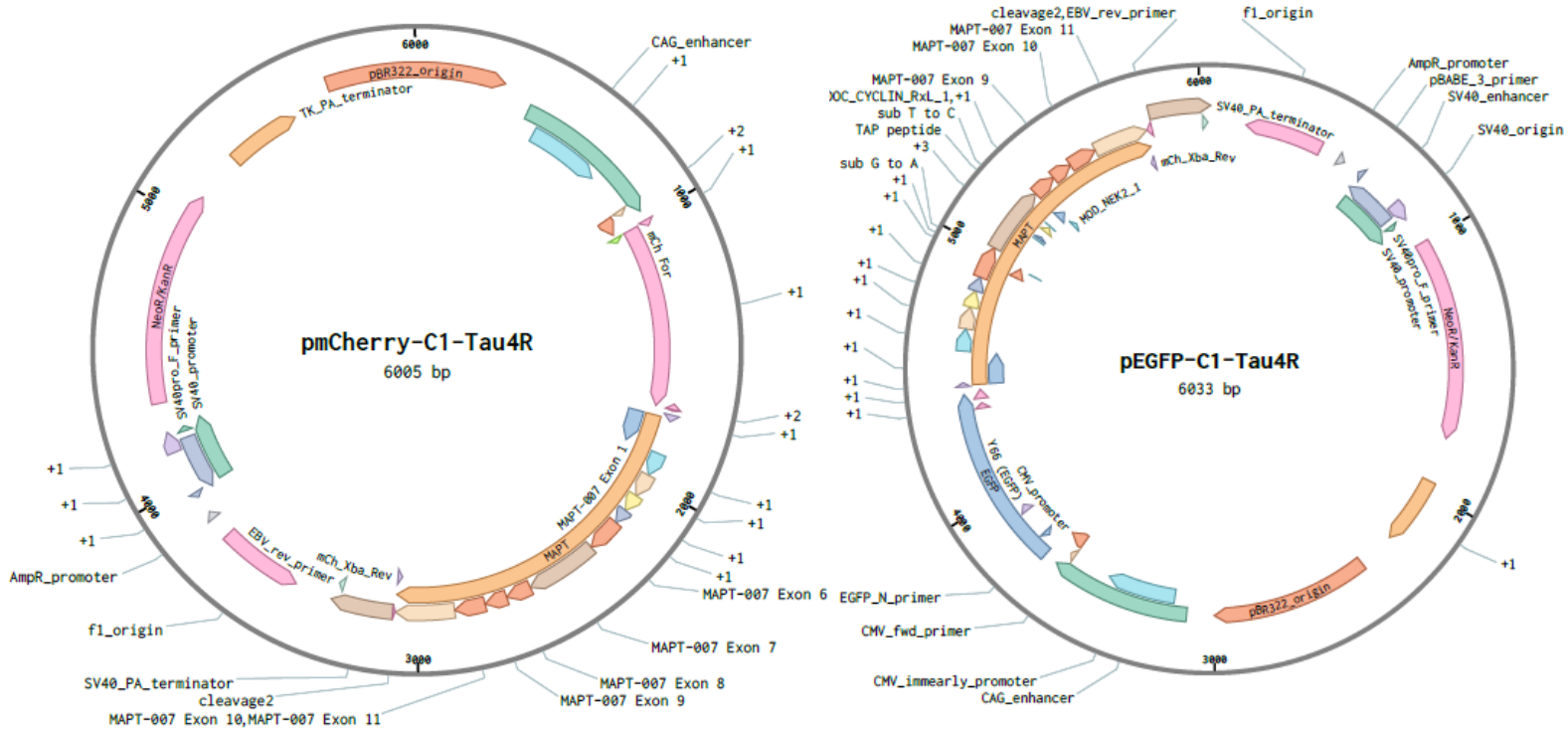

Supplement: S2 Fig — pmCherry-C1 and pEGFP-C1 vectors (A), human Tau3R (B) and 4R (C) expressing plasmids based on pmCherry-C1 and and pEGFP-C1 vectors. The plasmid maps were constructed with Benchling platform (www.benchling.com). (PDF) [file pone.0213666.s003.pdf]

S3 Fig

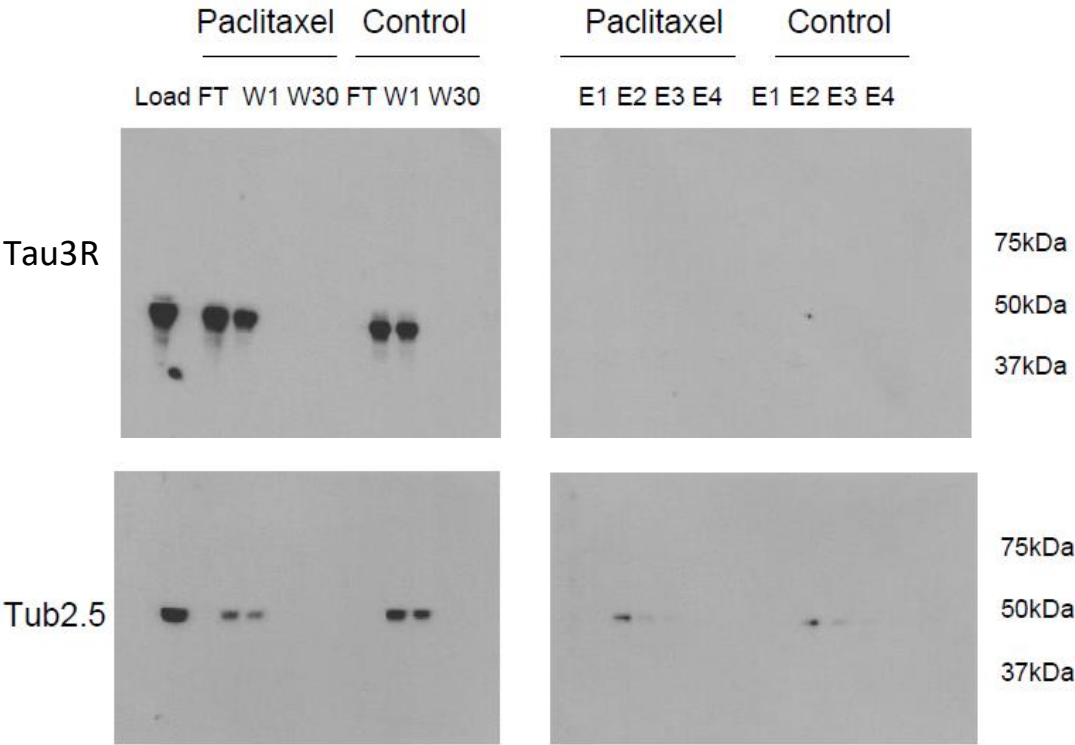

Supplement: S3 Fig — The gel lanes contain the protein loaded (load) flow-through (FT) PBS wash, pH7.5 (W1-30) and acid elutes (E1-E4) from the columns linked to eight-amino-acid inactive peptide VLGGGCALL P (has previously shown no microtubule-related neuroprotective activity [28]) that were incubated with brain extracts and DMSO in the absence and presence of paclitaxel. Western blotting analysis with anti Tau RD3 detected Tau3R presence in the loaded material, column flow-through and column wash, but did not detect Tau3R in the acid elution fractions of both the columns. In contrast, tubulin antibodies—Tub2.5 identified tubulin-like bands also in the elution fractions with no apparent influence of paclitaxel treatment. (PDF) [file pone.0213666.s004.pdf]

S4 Fig

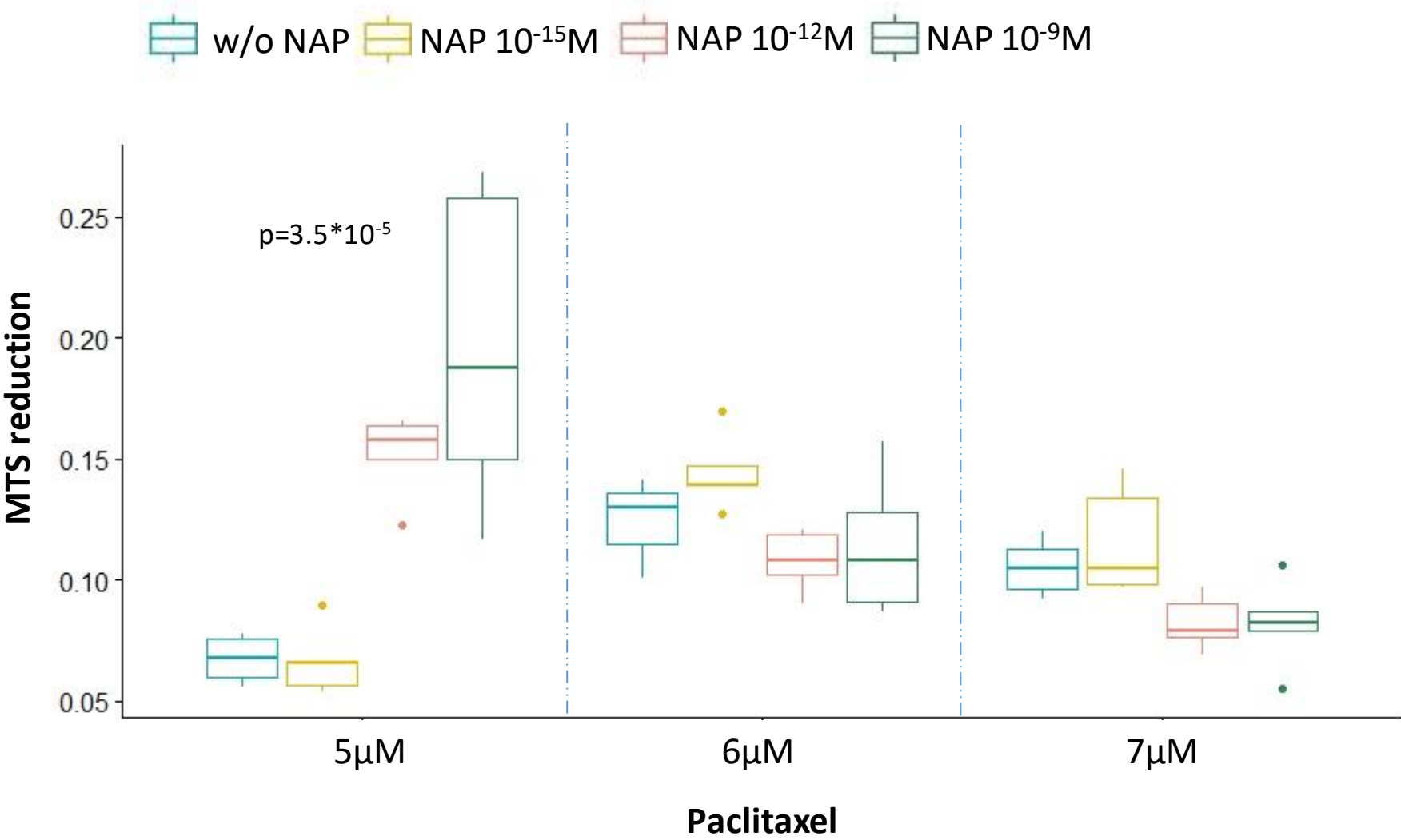

Supplement: S4 Fig — Examination of the effect of the two factors (Paclitaxel and NAP) showed that NAP had a significant effect only for the lower paclitaxel dose (D = 5). The indicated p-value is based on one-way ANOVA for this group; figure was generated using R. P-values of two-way ANOVA: paclitacel—0.00257; NAP—0.01093; paclitaxel:NAP interaction—3.58e-10. (PDF) [file pone.0213666.s005.pdf]

S5 Fig

IP: GFP

IB:  $\alpha$ -Tubulin

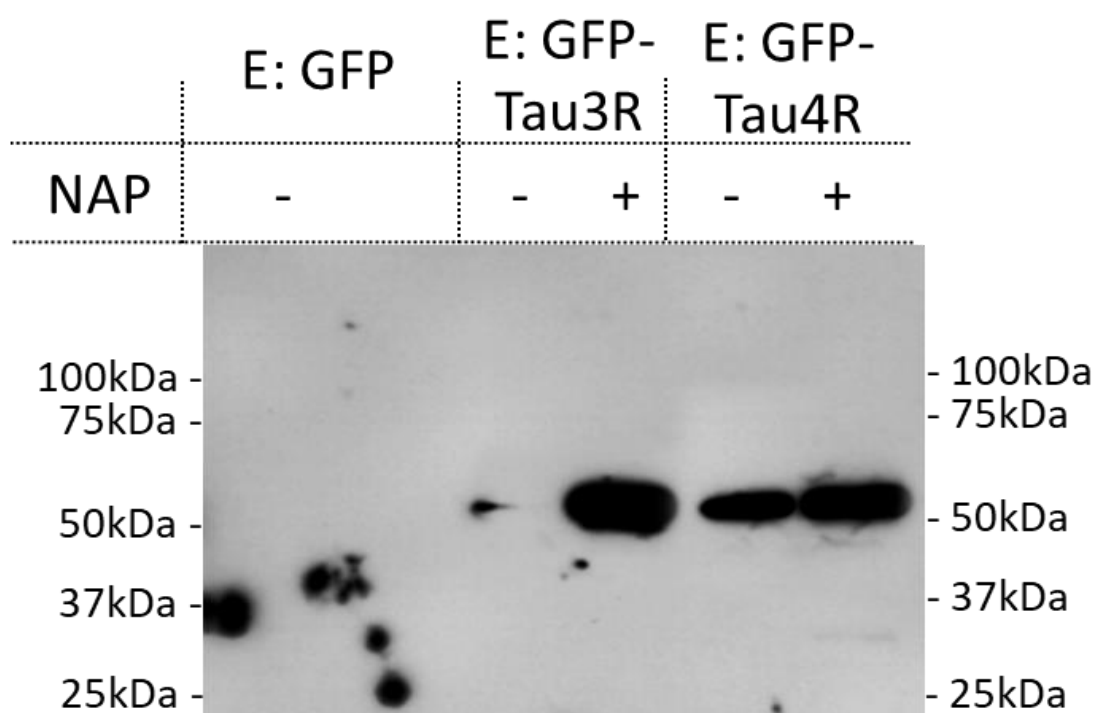

Supplement: S5 Fig — α-Tubulin. Differentiated human neuroblastoma SH-SY5Y cells were over-expressed with GFP-Tau3R or GFP-Tau4R. Cells with GFP expression were used as negative control. Immunoprecipitation (IP) of GFP, GFP-Tau3R and GFP-Tau4R in the presence and absence of NAP was done with GFP antibody. Elution fractions (E) analyzed by immunoblotting (IB) with tubulin antibody. (PDF) [file pone.0213666.s006.pdf]

**S1 Table**

**
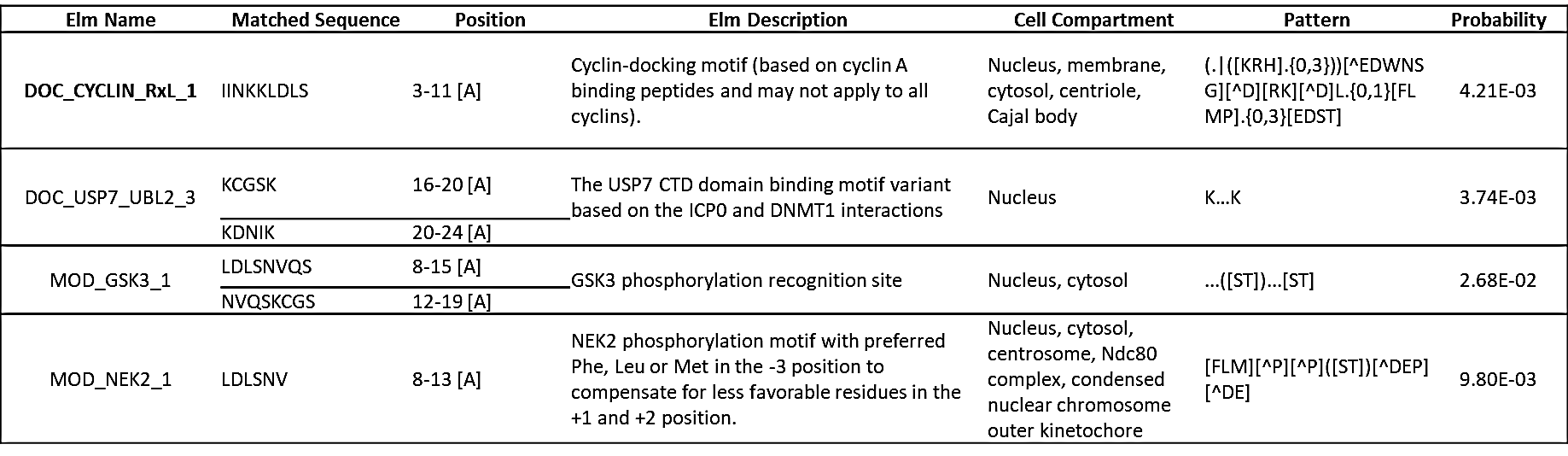
**

Supplement: S1 Table — ELM analysis [30] predicted functional motifs of the translation sequence of spliced exon 10 (VQIINKKLDLSNVQSKCGSKDNIKHVPGGGS) of Tau isoform 2 (NP_005901). DOC_CYCLIN_RxL_1 motif appeared only once in full Tau sequence. (DOCX) [file pone.0213666.s007.docx]
